# Correlates and determinants of transport-related physical activity among adults: an interdisciplinary systematic review

**DOI:** 10.1186/s12889-022-13937-9

**Published:** 2022-08-10

**Authors:** Jack T. Evans, Hoang Phan, Marie-Jeanne Buscot, Seana Gall, Verity Cleland

**Affiliations:** 1 Menzies Institute for Medical Research, University of Tasmania, 17 Liverpool St., 7000 Hobart, Australia; 2 Adjunct Associate Professor, School of Clinical Sciences, Monash University, Melbourne, Australia; 3 Honorary Fellow, School of Exercise and Nutrition Sciences, Deakin University, Geelong, Australia

**Keywords:** Transportation, Exercise, Physical activity, Risk factors, Behaviours, Adult, Systematic review

## Abstract

**Introduction:**

Transport-related physical activity (TRPA) has been identified as a way to increase physical activity due to its discretionary and habitual nature. Factors thought to influence TRPA span multiple disciplines and are rarely systematically considered in unison. This systematic review aimed to identify cross-sectional and longitudinal factors associated with adult TRPA across multiple research disciplines.

**Methods:**

Using four electronic databases, a systematic search of English, peer-reviewed literature from 2010 – 2020 was performed. Studies quantitatively examining factors associated with the outcome of adult TRPA were eligible.

**Results:**

Seventy-three studies (*n* = 66 cross-sectional; *n* = 7 longitudinal) were included, cumulatively reporting data from 1,278,632 observations. Thirty-six factors were examined for potential association with TRPA and presented in a social-ecological framework: individual (*n* = 15), social (*n* = 3), and environmental (*n* = 18). Seven factors were found to be consistently associated with higher adult TRPA: lower socio-economic status, higher self-efficacy, higher social normalization, lower distance of travel, higher destination concentration, more streetlighting, and higher public transportation frequency with a greater number of terminals near route start and endpoints.

**Conclusions:**

This is the first comprehensive compilation of the correlates and determinants of adult TRPA. Seven individual, social, and environmental factors demonstrated consistent associations with TRPA. Models formed using these factors may facilitate more effective promotion of TRPA. There is a lack of longitudinal studies as well as studies assessing cognitive/attitudinal and social factors, highlighting gaps for further research. Those developing policies and strategies targeting TRPA need to consider a range of factors at the individual, social, and environmental level to maximise the likelihood of effectiveness.

**Supplementary Information:**

The online version contains supplementary material available at 10.1186/s12889-022-13937-9.

## Introduction

Physical inactivity is the fourth leading cause of morbidity and mortality internationally, with an economic burden estimated to exceed INT$67.5 billion in 2013 alone [1]. Physical activity (PA) remains under-utilized by the general population as a means of health improvement [2]. Recent international estimates show that one in four adults do not meet the World Health Organization minimum recommendation of 150 min of moderate intensity PA a week [3]. Given the prevalence of physical inactivity and the role of PA in the prevention and management of chronic disease outcomes [4, 5], the promotion of PA has become a global health priority [3].

Physical activity can be accumulated across four key settings or domains: leisure-time (e.g. sport, exercise), transport (e.g., walking or cycling for transport), domestic (e.g., home or yard maintenance), and occupational PA (e.g., activity undertaken as part of employment). Transport-related PA (TRPA) (also known as active commuting), has been highlighted as a potential means for the increase of PA and improvement of population health [6]. TRPA comprises of healthy active travel behaviours such as walking or cycling for means of commute. This is both as a sole means of transportation or in combination with public or private transport. Both TRPA and leisure-time PA may be considered predominantly discretionary(those with private vehicles have choice as to whether they undertake private, or public and active transport) [7], and hence more amenable to intervention. When compared to leisure-time PA, TRPA remains comparatively understudied and as such represents an important opportunity to research and gain an understanding of how PA may be further integrated into daily life.

TRPA is associated with reduced all-cause mortality [8, 9], lowered risk of cardiovascular disease [10], and some cancers [11], independent of total PA [12]. Moreover, the undertaking of TRPA, independent of other domains of PA, has the potential to provide a substantial increase in total PA levels [13]. For example, people who used public transport in the United States accumulated an additional 30 min of PA each day via the walk to and from public transport stops compared to people who did not use public transport [14, 15]. Similarly, a study of German adults found 48% of participants achieved the global PA recommendation of 150 min per week solely via their active commute [16]. While many factors are thought to influence an individual’s engagement in TRPA, these variables stem from differing disciplines (i.e., environmental, socio-ecological, behavioural, and health/medicine-related [17–19]) that are rarely considered in unison. To date there has not yet been a systematic compilation or critical analysis of the factors associated with TRPA spanning multiple disciplines of study. The organisation of these factors within a theoretical framework would provide a structured approach to understanding associations with TRPA. The use of a social-ecological model allows for the categorisation of factors into individual (e.g., age, smoking status, income, self-efficacy), social (e.g., cohesion, normalisation), organisational (e.g., workplace TRPA incentives), environmental (e.g., distance, destination, traffic), and policy-based levels (e.g., promotion of PA guidelines and implementation of interventions). Therefore, this systematic review aimed to identify the cross-sectional correlates and longitudinal determinants of adult TRPA across multiple disciplines of research and structure them within a social-ecological framework.

## Methods

This systematic review was registered on the PROSPERO International Prospective Register of Systematic reviews (Registration Number: CRD42020184487) and executed in compliance with the guidelines of the Meta-Analyses and Systematic Reviews of Observational Studies (MOOSE) and Preferred Reporting Items for Systematic Reviews and Meta-Analyses (PRISMA) statements [20, 21]. A full protocol may be requested from the authors.

### Literature search

J.E. conducted an independent literature search of four online databases (Web of Science Core Collection, Scopus, Medline, and Embase via Ovid) for published journal articles examining factors associated with adult TRPA outcomes across the last decade (2010 – 2020). Landmark journal articles were first screened to derive terms for search inclusion.

Using terms derived in combination with MeSH (Medical Subject Heading) terms, search filters were included to restrict results to peer-reviewed journal articles published in the English language. Literature search results were imported to Covidence (systematic review management software) [22] where duplicates were first removed, then screening performed. Reference lists of relevant publications were searched for additional studies not returned via database screening.

### Study inclusion criteria

Studies were included within this systematic review provided they met the criteria of: (i) publication as a full-length article in a peer-reviewed English language journal, (ii) adult participants (aged ≥ 18 years) with no restriction on sex, ethnicity, or health status, (iii) reporting adult TRPA via self-report or objective measurement either as a primary or secondary outcome, and (iv) quantitatively examined factors cross-sectionally or longitudinally associated with the outcome of adult TRPA. For the purposes of this study, sex and gender identity were analysed in conjunction with one another. Failure of a study to meet any of these inclusion criteria resulted in its exclusion from this review.

### Data extraction and analysis

All search results were independently screened for inclusion by J.E. and H.P. Title/abstract content were first screened, with articles then considered for inclusion undergoing secondary screening via assessment at the full-text level. Final inclusion conflicts were discussed by the two reviewing authors. Any unresolved inclusion/exclusion dispute was moderated by a third author (V.C.). Paper characteristics including country of study, study design, participant characteristics, outcome measure, and results were extracted by J.E. and H.P.

### Quality assessment

The quality of studies included was assessed via a modified Newcastle – Ottawa Scale [23] (Additional file 1). In this scale the quality of studies and risk of bias was assessed across three categories: selection of participants and sample representativeness, the comparability of participants, and the assessment of outcome. Studies were then categorized as good, fair, or poor quality. Studies with a ‘poor’ quality rating were excluded from the final analysis.

## Results

### Study characteristics

**Fig. 1:**
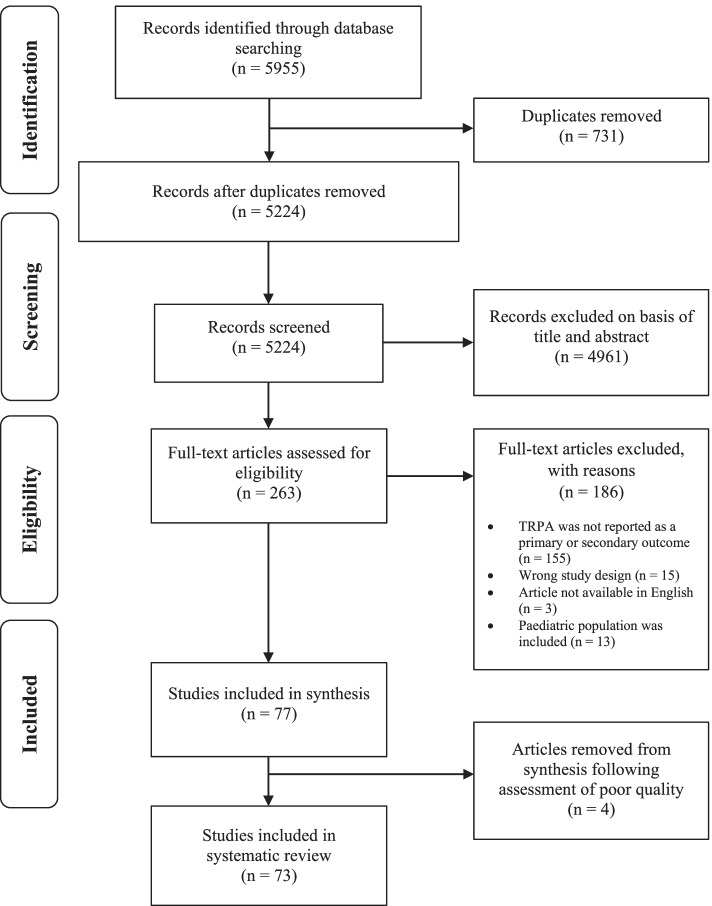
PRISMA flow chart for the selection of studies

The search of online databases yielded 5955 studies. Shown within the PRISMA flowchart of Fig. 1, 731 duplicates were removed with 5224 abstracts and 263 full texts screened for inclusion. After removing 190 irrelevant articles, 77 studies remained. Quality assessment determined the methodology of four of these studies to be of poor quality (see below for more details), and resultantly exclusion occurred. This yielded a total of 73 studies for inclusion in this systematic review (Fig. 1). Of these 73 studies, 35 assessed TRPA using IPAQ or GPAQ questionnaires, both of which ask about commuting for any purpose. Of the remaining 38 studies, 34 used assessments of TRPA asked about commuting for any purpose (e.g., Belgian Aging Study questionnaire); four studies assessed TRPA to work only.

### Summary of included studies

Studies included within this review and outcome measures are summarised in Table 1. Seventy-three studies spanning 28 countries and 1,278,632 observations were represented. Study sample sizes ranged from 101 to 308,901 participants, with a mean gender distribution of 60.4% female. Only seven articles were found to longitudinally assess relationships with adult TRPA.

**Table 1:** Summary of included studies

Descriptors							Exposures		Outcomes		
Study ID Author, Year	Country	Study design	N	Sex, % Female	Age range ^*^, (years)				Description	Statistic	Assessment
Adams, 2013 [24]	United Kingdom	Cross-sectional	3516	54.9	≥ 18		Traffic safety, supportive infrastructure, local amenities (destinations), social order, street connectivity, general environmental quality		Dichotomous walking and cycling for transport: 0 min/week and > 0 min/week	Odds ratio	IPAQ
Adams, 2016 [25]	United Kingdom	Cross-sectional	1544	64.1	≥ 18		Route infrastructure, route lighting (streetlights). Route free of litter/ graffiti (aesthetics), pleasant walking, convenient public transport		Dichotomous walking for transport: 0 min/week and > 0 min/week	Odds ratio	Transport and physical activity questionnaire
Adams, 2017 [26]	United Kingdom	Cross-sectional	1189	65.6	≥ 18		Age, education, ethnicity, vehicle access, physical activity, work-related physical activity, distance, free car parking at work, work hours, work pattern, occupation, psychosocial factors (attitude, behavioural control, intention, social norms, colleague support), perceived barriers		Dichotomous walking for transport: 0 min/week and > 0 min/week	Odds ratio	IPAQ-S
Adlakha, 2015 [27]	United States	Cross-sectional	2015	-	21–65		Large selection of fresh fruits and vegetables; Opportunities to purchase fast food; presence of healthy restaurants; 10–15-min walk to a transit stop; sidewalks on most streets; shops, stores, or markets; facilities to bicycle; recreation facilities; crime rates; traffic; See people being physically active		Dichotomous TRPA: < 150 min/week and ≥ 150 min/week	Odds ratio	IPAQ-S
Adlakha, 2017 [28]	India	Cross-sectional	370	54.2	18–65		Age, sex, marital status, religion, education, income, employment, density, land-use mix, street connectivity, infrastructure for walking/bicycling, aesthetics, safety from traffic and crime		Dichotomous TRPA: < 150 min/week and ≥ 150 min/week	Odds ratio	IPAQ-L
Aliyas, 2019 [29]	Iran	Cross-sectional	1833	50.8	18–70		Age (> 30 years), sex, education, marital status, occupation (employed), vehicle access, safety, crime, social ties, collective efficacy (social modelling)		Dichotomous TRPA level: < 60 min/week and ≥ 60 min/week	Odds ratio	IPAQ-L
Aliyas, 2020 [30]	Iran	Cross-sectional	1132	50.7	18–65		Age, sex, marital status, education, years at current address, number of children < 12yo, safety		Dichotomous average walking for transport: < 30 min/week and ≥ 30 min/week	Odds ratio	IPAQ-L
Amorim, 2010 [31]	Brazil	Cross-sectional	972	57.0	20–69		Sidewalks, green-space, garbage accumulation, sewage presence, traffic impact on walk/ride, crosswalks, exhaust fumes, streetlights at night, crime, sports events, weather		Dichotomous TRPA: 0–149 min/week and ≥ 150 min/week	Prevalence ratio	IPAQ-L
Barr, 2019 [32]	Australia	Cross-sectional	4913	46.4	≥ 18		Local and regional accessibility measures (walkability)		Walking for transport: min/week	Regression coefficient	Accelerometer
Barranco-Ruiz, 2019 [33]	Chile	Cross-sectional	496	68.0	≥ 18		Age, distance, socio-economic status, existing physical activity patterns		Commute mode: active or passive	Odds ratio	Questionnaire
Bauman, 2011 [34]	Australia, China, Fiji, Malaysia, Nauru, Philippines	Cross-sectional	173,206	54.1	18–64		Age, sex, education, income, area (urban vs rural)		High TRPA: Australia (NA); China (≥ 30 min/day); Fiji (always or usually); Malaysia (≥ 3 days/week and accumulating ≥ 3000 MET-min/week); Philippines (top quartile)	Odds ratio	Survey
Bopp, 2014 [35]	United States	Cross-sectional	706	100	≥ 18		Age, marital status, race/ethnicity, number of dependants, income, education, body mass index, chronic disease, self-reported health, employment, vehicle access, self-efficacy, physical activity behaviours, social norms, social modelling, distance, infrastructure, sidewalks, traffic, safety, weather		Dichotomous active commute: 0 trips/week and ≥ 1 trip/week	Odds ratio	Survey
Bopp, 2019 [36]	United States	Longitudinal	204	60.7	≥ 18		Body mass index, stress level, depressive symptoms, existing physical activity level, distance, employment		Dichotomous TRPA (mins/week): top quartile = high TRPA	Odds ratio	GPAQ
Borchardt, 2019 [37]	Brazil	Cross-sectional	1429	57.0	18–96		Density, income, destinations, infrastructure, aesthetics, safety, proximity to coast, infrastructure		Dichotomous walking or cycling for transport: Yes = 10 consecutive minutes in previous 7 days; No = no TRPA exceeding 10 consecutive minutes	Prevalence ratio	IPAQ-L
Brondeel, 2016 [38]	France	Cross-sectional	21,332	-	35–83		Age, sex, employment, education, income, distance to public transport, vehicle access, transport behaviours, commute trip characteristics, size of parks, destinations, intersections (connectivity), population density		Transport-related moderate to vigorous physical activity (min/day)	Incidence risk ratio	Accelerometer
Cerin, 2013 [39]	Canada	Cross-sectional	484	58.0	≥ 65		Destination diversity and prevalence, infrastructure, safety		Walking for transport (min/week)	Anti-logarithm of regression coefficient	IPAQ-L
Chudyk, 2017 [40]	Canada	Cross-sectional	161	63.4	74.3 (6.2)		Age, sex, marital status, vehicle access, pet ownership, Street Smart Walk Score (walkability), aesthetics, safety, body mass index, gait speed, comorbidities (health), individual enjoyment / attitudes (physical activity behaviours), social cohesion		Walking for transport: any or none; frequency (trips/week)	Regression coefficient	Community Healthy Activities Model Program for Seniors survey
Cleland, 2010 [41]	Australia	Cross-sectional	4349	100	18–45		Age, area (rural vs urban), education, employment, marital status, number of children (dependants), health and health behaviours (weight status, pregnancy, illness, smoking), self-efficacy, physical activity behaviours (enjoyment, intention, outcome expectancies), childcare, family and friend support, pet ownership, social cohesion, safety, aesthetics, walking environment		Categorical TRPA: low (0–29 min/week), medium (30–149 min/week), or high (> 150 min/week)	Odds ratio	IPAQ-L
Cleland, 2012 [42]	Australia	Cross-sectional	3667	100	18–45		Self-efficacy, enjoyment, outcome expectancy, intentions, skills, childcare availability, family support, friends support, dog ownership, safety, aesthetics, walking environment		Categorical TRPA: low (1–89 min/week), medium (90–209 min/week), or high (≥ 210 min/week)	Odds ratio	IPAQ-L
Cleland, 2020 [43]	Australia	Longitudinal	1480	100	18–46		Age, country of birth, English spoken at home, education, income, number of children, health, body mass index, smoking status, pregnancy, menopause, physical activity enjoyment, family support, childcare availability, existing physical activity behaviours		TRPA (min/week)	Odds ratio	IPAQ-L
Corseuil Giehl, 2017 [44]	Brazil	Cross-sectional	1705	63.9	≥ 60		Sidewalks, crosswalk, aesthetic, streetlighting, safety, pet ownership, parks/recreational destinations		Categorical walking for transportation: none, 10–149 min/week, ≥ 150 min/week	Odds ratio	IPAQ-L
Dedele, 2019 [45]	United Kingdom	Cross-sectional	1111	57.7	≥ 18		Age, sex, marital status, number of dependants, educational, employment, income, vehicle access, body mass index, chronic disease (health), smoking / alcohol consumption (health behaviours), physical activity and mobility behaviour, socioeconomic status		Dichotomous TRPA: 0–29 min/day and ≥ 30 min/day	Prevalence, odds ratio	GPAQ
Del Duca, 2013 [46]	Brazil	Cross-sectional	1720	54.4	35–74		Age, sex, skin colour, marital status, education, family income		Dichotomous TRPA: inactivity and active	Prevalence ratio	Surveillance System of Protective and Risk Factors for Chronic Diseases
de Matos, 2018 [47]	Brazil	Cross-sectional	15,105	54.4	35–74		Age, ethnicity, dependent relatives, weight/anthropometric status, socio-economic status, traffic, safety, walkability, opportunities for physical activity		Categorical TRPA: inactive (< 10 min/week), insufficiently active (10–149 min/week), physically active (≥ 150 min/week)	Relative risk ratio	IPAQ-L
Durand, 2017 [48]	United States	Cross-sectional	65,905	52.5	47.2 (30.8)		Daily measures of mean hourly temperature (degrees Fahrenheit), relative humidity (%), wind speed (miles per hour) and total daily precipitation (inches; includes snow and rain)		TRPA trip duration (min)	Regression coefficient	Travel diary
Eichinger, 2015 [49]	Austria	Cross-sectional	904	42.2	18–91		Sex, distance, supportive infrastructure, connectivity, traffic and crime safety, pleasant environment, presence of trees (green space) social cohesion / support, social modelling, total physical activity		TRPA: MET min/week	Regression coefficient	IPAQ-L
Falconer, 2017 [50]	United Kingdom	Cross-sectional	6896	35.1	≥ 35		Age, sex, deprivation, household income, health, distance, commute frequency, population density, air pollution, traffic density, proximity to major road, distance to major road		Dichotomous: active commute and no active commute	Odds ratio	IPAQ-L
Freeland, 2013 [14]	United States	Cross-sectional	308,901	50.8	≥ 18		Age, sex, ethnicity, education, household income, race /ethnicity, vehicle access / ownership, employment status, urban size / density		Dichotomous walking for transport: < 30 min/day and ≥ 30 min/day	Odds ratio	National Household Travel Survey
Ghani, 2018 [51]	Australia	Cross-sectional	11,035	-	40–65		Age, residential density, street connectivity, land-use mix		Dichotomous walking for transport: "none" (0 min/week) and "any" (1–840 min/week)	Regression coefficient	Single question
Gul, 2019 [52]	Pakistan	Cross-sectional	1042	33.3	18–65		Age, sex, employment status, education, mode of transportation, marital status, neighbourhood type (gated / non-gated)		Practical walking: MET min/week	T-test, Pearson chi-square	NPAQ
Kwasniewska, 2010 [53]	Poland	Cross-sectional	7280	48.5	20–74		Age, place of residence, education, income, marital status, smoking status, leisure-time physical activity, occupational physical activity		Categorical TRPA: 0 min/day; 1–14 min/day; 15 to 29 min/day; ≥ 30 min/day; and active or inactive	Odds ratio	Questionnaire
Li, 2020 [54]	United States	Cross-sectional	2848	60.0	≥ 18		Age, sex, education, income, race/ethnicity, years lived in neighbourhood, walkability, safety, aesthetics, financial cost, and time trade-off		Walking for transport (min/week) and willingness to walk for transport	Structural Equation Model	Survey
Liao, 2017 [55]	Taiwan	Cross-sectional	1068	50.8	20–64		Public bicycle use		Dichotomous TRPA: < 150 min/week and ≥ 150 min/week	Odds ratio	IPAQ-L
Lima, 2017 [56]	Brazil	Cross-sectional	602	37.7	≥ 18		Age, sex, socio-economic level, education, physical activity behaviours, active/sedentary status		TRPA (min/week) and transportation mode	Students t-test	IPAQ-S
Lopes, 2018 [57]	Brazil	Cross-sectional	1419	63.6	≥ 18		Age, sex, marital status, socioeconomic status, nutritional status, self-rated health/ quality of life, perceived neighbourhood crime, motor vehicle access, days of public transport use per week, land use, streetscape, aesthetics, sidewalks, streets, social environment		Categorical walking for transport: ≥ 10 min/week and ≥ 150 min/week; Bicycling for transport: ≥ 10 min/week	Prevalence ratio	IPAQ-L
Lu, 2017 [58]	China	Cross-sectional	1078	-	18–65		Age, sex, education, population density, income, intersection density, land-use mix		Walking for transport (min/week)	Regression coefficient	IPAQ-L
Mackenbach, 2016 [59]	New Zealand	Cross-sectional	481	46.8	20–65		Income, population density, housing density, apartment density, land-use mix, public transport access and frequency, job accessibility, parking price, area deprivation, walkability		TRPA: Trips with an active mode ≥ 10 min	Odds ratio	New Zealand Household Travel Survey
Malambo, 2017 [60]	South Africa	Cross-sectional	671	76.0	35–70		Land-use mix, street connectivity, infrastructure, aesthetics, safety (traffic/crime), urban / rural status		Dichotomous TRPA: < 150 min/week and ≥ 150 min/week	Odds ratio	IPAQ-L
Matsushita, 2015 [61]	Japan	Cross-sectional	3269	49.6	30–59		Age, sex, household income, education, employment, number of motor vehicles, body mass index		Dichotomous TRPA: inactive (< 10 min/week) and active (≥ 10 min/week)	Odds ratio	GPAQ
Mertens, 2019 [62]	Belgium	Longitudinal	438	54.1	≥ 65		Age, education, baseline transport-related physical activity, self-efficacy, neighbourhood social trust, neighbourhood social diversity, land-use mix, infrastructure, aesthetics, safety		Walking for transport: ≥ 10 min/week (engagement)	Odds ratio	IPAQ-L
Molina-García, 2014 [63]	Spain	Cross-sectional	518	59.7	≥ 18		Age, sex, socio-economic status, residence type (home or campus), distance, main transport mode		TRPA: MET min/week and commute mode	t-test, ANOVA	Survey
Mumford, 2011 [64]	United States	Longitudinal	101	67.0	≥ 18		Neighbourhood density, land-use mix		Walking for transportation: mins/week and days/week	Odds ratio	Survey
Nathan, 2014 [65]	Australia	Cross-sectional	323	68.1	76.9 (7.3)		Aesthetics, safety, physical barriers, walkability, infrastructure		Dichotomous walking for transport: < 60 min/week and ≥ 60 min/week	Odds ratio	Community Healthy Activities Model Program for Seniors survey
Nordfjærn, 2019 [66]	Norway	Cross-sectional	441	53.0	23.1 (4.8)		Age, sex, campus (area density), ascription of responsibility, awareness of consequences, safety, priorities of physical activity, convenience, duration / distance, vehicle access		Active transportation use	Regression coefficient	Questionnaire
Padrão, 2012 [67]	Mozambique	Cross-sectional	3211	-	25–64		Age, sex, education, physical activity behaviours, urban / rural status		TRPA: ≥ 60 min/day	Prevalence ratio	GPAQ
Panter, 2014 [68]	United Kingdom	Longitudinal	655	69.0	18–69		Pleasant walk environment, convenient public transport, traffic, safety, convenient routes		Change in TRPA (min/week); Uptake of TRPA	Odds ratio and regression coefficient	Survey
Panter, 2011 [69]	United Kingdom	Cross-sectional	1142	68.0	42.3 (11.4)		Sex, vehicle access, distance, public transport, traffic, routes, safety, urban / rural status, vehicle use (intent, attitude, norms, habit)		Walking for transport: no engagement and any engagement; Cycling for transport: < 150 min/week and ≥ 150 min/week	Odds ratio	Survey
Panter, 2011 [69]	United Kingdom	Longitudinal	1279	53.1	49–80		Age, sex, body mass index, employment, habit, control, intent, attitude, subjective norm, social support, distance, perceived environment, residence type, socio-economic deprivation, land-use mix, access, street connectivity, infrastructure, aesthetics, safety, urban/rural status, density, streetlights, connectivity, sidewalks, walkability		Commute mode: active or non-active	Odds ratio	EPAQ2
Pelclova, 2013 [70]	Czech Republic	Cross-sectional	2839	50.1	≥ 50		Residential density, land use-mix, street connectivity, infrastructure, aesthetics, safety		Walking for transport: < 30 min/day and ≥ 30 min/day	Odds ratio	IPAQ-L
Perchoux, 2017 [71]	France	Cross-sectional	23,432	100	≥ 18		Occupation intensity, leisure-time physical activity, transportation type, destinations, infrastructure, aesthetics, social norms, social modelling		TRPA (hours/week) determining cluster allocation	Odds ratio	STAQ
Quinn, 2017 [72]	United States	Cross-sectional	152,573	48.5	≥ 18		Age, sex, education, race, income, urban / rural status, employment, distance / duration, employment start time		TRPA: non-active (< 10 min/trip) and active (≥ 10 min/trip)	Odds ratio	Interview
Reilly, 2013 [73]	United States	Cross-sectional	387	96.0	18–39		Age, sex, education, income, marital status, birthplace, length of US residency, health insurance status, physician communication		TRPA: no engagement and any engagement	Odds ratio	GPAQ
Ryan, 2018 [74]	Canada	Cross-sectional	5180	52.5	20–64		Age, sex, income, education, urban / rural status, health, smoking status, body mass index, aboriginal language, spirituality		Categorical walking for transportation: < 1 h/week, 1–5 h/week, > 5 h/week	Odds ratio	Aboriginal Peoples Survey
Saris, 2013 [75]	Netherland	Cross-sectional	622	54.2	≥ 18		Age, sex, ethnicity, body mass index, neighbourhood status score (infrastructure, traffic, safety)		TRPA: walking and cycling for transport (mins/week)	Regression coefficient	SQUASH
Shimura, 2012 [76]	Australia	Longitudinal	504	54.0	50–65		Neighbourhood walkability		Changes in walking for transport: min/day	Regression coefficient	IPAQ-L
Simons, 2017 [77]	Belgium	Cross-sectional	224	56.0	18–26		Self-efficacy, social support, social norms, social modelling, perceived benefits, perceived barriers, land-use mix, street connectivity, walking and cycling facilities, aesthetics, work facilities, distance, density, safety, education level		Transport mode, TRPA duration (min/day), TRPA frequency (days/week)	Odds ratio	IPAQ-L
Slater, 2016 [78]	United States	Cross-sectional	311	58.5	18–45		Age, sex, body mass index, education, income, marital status, smoking, cancer diagnosis, vehicle access, leisure/work/household physical activity, environmental barriers, planning/psychosocial barriers, safety barriers, health barriers, walkability		TRPA: no engagement and any engagement	Odds ratio	IPAQ based questionnaire
Thern, 2015 [79]	Sweden	Cross-sectional	432	52.0	20–52		Ethnicity, pet ownership, residential area, environment, alcohol consumption, outdoor recreational physical activity, indoor physical activity		Dichotomous TRPA: active (if a person walked or cycled ≥ 15 min, one-way to school or work) and non-active (if a person walked or cycled for < 15 min, one-way to school or work)	Odds ratio	Swedish Survey of Living Conditions
Van Cauwenberg, 2012 [80]	Belgium	Cross-sectional	48,879	55.7	≥ 60		Age, sex, education, income, functional limitations, distance, destinations, public transport, infrastructure, sidewalks, intersections, safety, streetlighting, aesthetics, greenness, urban / rural status,		Dichotomized walking and cycling for transport: "almost daily walking for transportation" and "less than almost daily walking for transportation" or "almost daily cycling for transportation" and "less than almost daily cycling for transportation"	Odds ratio	Belgian Aging Study questionnaire
Van Cauwenberg, 2013 [81]	Belgium	Cross-sectional	50,685	55.5	≥ 60		Age, sex, marital status, functional limitations, educational, income, area (urban / semi-urban), Environmental index (absence of high curbs, destinations, benches, crossings, bus stops, street lighting, safety from crime), distance		Dichotomized walking for transport: "almost daily walking for transportation" and "less than almost daily walking for transportation"	Odds ratio, predicted probability	Belgian Aging Study questionnaire
Van Cauwenberg, 2014 [82]	Belgium	Cross-sectional	24,875	55.6	≥ 65		Frequency of contact with neighbours, satisfaction of contact with neighbours, neighbour social support, community members, formal community engagement		Dichotomized walking for transport: "almost daily walking for transportation" and "less than almost daily walking for transportation"	Odds ratio	Belgian Aging Study questionnaire
Van Dyck, 2010 [83]	Belgium	Cross-sectional	1200	52.1	20–65		Walkability		Walking and cycling for transportation (min/week)	Regression coefficient	IPAQ-L
Van Dyck, 2013 [84]	Belgium	Cross-sectional	4139	100	18–46		Aesthetics, physical activity environment, personal safety, neighbourhood social cohesion		Walking for transportation (min/week)	Regression coefficient	IPAQ-L
van Heeswijck, 2015 [85]	Canada	Cross-sectional	37,165	52.0	20–89		Density, land-use mix, greenness, intersection density		Dichotomous TRPA: "sedentary" and "active" commute	Odds ratio	Questionnaire
Veitch, 2013 [86]	Australia	Cross-sectional	319	65.3	55.9 (15.4)		Park visitation		Categorical TRPA: low (0–90 min/week), medium (91–275 min/week), high (≥ 276 min/week)	Odds ratio	IPAQ-L
Wasfi, 2013 [87]	Canada	Cross-sectional	6913	57.0	33.6 (12.4)		Age, sex, income, travel behaviour (type, frequency, distance), social characteristics (education), population density, destination density, intersections		Total walking distance/day for commute (metres)	Regression coefficient	Geographic Information System
Weber Corseuil, 2012 [88]	Brazil	Cross-sectional	1656	63.9	60–102		Streetlighting, safety		Dichotomous TRPA: < 150 min/week and ≥ 150 min/week	Prevalence ratio	IPAQ-L
Wilson, 2012 [89]	Australia	Cross-sectional	10,745	55.7	40–65		Density, hilliness, tree coverage, bikeways, streetlights, river or coast, public transport, shop, land-use mix		Categorical walking for transportation: 0 min/week, 1–59 min/week, 60–149 min/week, ≥ 150 min/week	Odds ratio	Questionnaire
Witten, 2012 [90]	New Zealand	Cross-sectional	2033	57.2	20–65		Dwelling density, street connectivity, land-use mix, streetscape, neighbourhood destinations accessibility index		TRPA (min/week) transformed to have a standard deviation of one	Odds ratio	IPAQ-L
Yang, 2017 [91]	United Kingdom	Longitudinal	1143	69.0	40–79		Distance, streetlighting, walkability, main or secondary road on route		Commute mode: "active" and "passive" commuters; Categorical change in commuter mode over time	Odds ratio	EPAQ2
Yang, 2020 [54]	United States	Cross-sectional	125,819	-	≥ 18		Age, sex, ethnicity, education, income, employment, neighbourhood population density, driver status, vehicle access		TRPA: trips/week	Adjusted means	National Household Travel Survey
Yu, 2020 [92]	United States	Cross-sectional	109,617	49.6	≥ 18		Sex, race, education, income, population density, number of vehicles, number of household members		Two dichotomous TRPA variables: (1) "did not walk" and "walked to/from transit to work"; (2) "walked ≥ 30 min/day to or from transit to work" and "walked < 30 min/day to or from transit to work"	Odds ratio	National Household Travel Survey
Zwald, 2014 [93]	United States	Cross-sectional	772	63.6	≥ 18		Age, sex, income, employment, public transport use, safety, traffic, sidewalks, destinations		Categorical walking for transportation: 0 min/week, 1–149 min/week, ≥ 150 min/week	Odds ratio	IPAQ-L

^*^ Where age range was not available, mean (standard deviation) was presented in place

- = No gender distribution reported

*EPAQ2* European prospective investigation into cancer and nutrition Physical Activity Questionnaire version two, *GPAQ* Global Physical Activity Questionnaire, *IPAQ (-L/-S)* International Physical Activity Questionnaire (-Long / -Short version), *NPAQ* Neighbourhood Physical Activity Questionnaire, *MET* Metabolic Equivalent of Task, *STAQ* Simpson-Troost Attitude Questionnaire, *SQUASH* Short Questionnaire to Assess Health-enhancing Physical Activity, *TRPA* Transport-related physical activity

### Quality and risk of bias assessment

Four articles were classified to be of lower quality and of higher bias risk when assessed using a modified Newcastle–Ottawa scale (Additional file 1). As such, they were excluded from this review. Assessments of quality are presented in the Quality Assessment Table, found within Additional file 2. Forty-one articles were deemed to be of fair quality and 32 were rated as good quality.

### Individual exposures

A number of individual level exposures from both biological and socio-economic origins were shown to be associated with adult TRPA. These associations are summarised in Table 2.

**Table 2:** Summary of relationships observed between exposures and transport-related physical activity outcomes

	Factor	Positive, n	Negative, n	No association, n
**Individual**	Age, older	3	12	24
	Sex, male	9	3	21
	Health			
	Self-report	5	-	6
	Weight / body mass index	1	4	6
	Health behaviours (smoking, alcohol)	-	3	4
	Ethnicity, white	1	4	9
	Marital status, partnered	1	4	7
	Number of dependants	1	2	4
	Pet ownership	1	1	3
	Employment, employed	1	4	7
	Income, greater	3	9	7
	Education, higher	6	4	15
	Socio-economic status, greater	-	9	1
	Motor vehicle			
	Access	-	7	7
	Parking	-	1	1
	Attitudes	-	1	-
	Self-efficacy	5	-	-
	Physical activity attitudes and behaviours	6	-	8
	Attitudes, behaviours, and beliefs	-	1	6
**Social**	Social cohesion	2	-	9
	Social modelling	3	-	4
	Normalisation	5	1	3
**Environmental**	Distance	-	11	1
	Destination	14	-	2
	Land-use mix	7	1	7
	Walkability	5	-	2
	Connectivity	10	1	10
	Supportive infrastructure	8	1	9
	Public transport	13	1	1
	Traffic	2	-	9
	Urban vs rural, urban	3	2	6
	Population and land density	5	1	10
	Green spaces	3	2	3
	Gradient, flat	1	-	3
	Park access / visitation	1	-	3
	Location, river/coast	2	-	-
	Aesthetics	12	2	11
	Weather	1	-	2
	Safety	13	5	12
	Streetlighting	7	-	3

### Physical, biological, and health and health behaviours

Age and sex were assessed among numerous studies, examined as either individual exposures, or covariates in multivariable models. Thirty-nine studies assessed the relationship between participant age and TRPA, fifteen of which found the relationship to be statistically significant. Increasing age was associated with decreasing odds of engaging in TRPA or a lower TRPA level in twelve studies [26, 29, 46, 51, 56, 57, 63, 66, 74–76, 87] including one longitudinal [76]. Conversely, a positive relationship between age and TRPA was observed among only three studies, in which women of lower socio-economic status [34, 41, 47] reported greater TRPA levels with higher age. Twenty-four studies found there to be no significant association between age and TRPA level [14, 30, 32, 33, 35, 40, 45, 50, 52–54, 58, 59, 62, 67–69, 72, 78, 82, 88, 92–94].

Significant differences in TRPA level by sex were noted among twelve of thirty-three studies. Nine articles reported male participants undertaking a greater amount of TRPA than women (three assessed walking and cycling combined into a single measure of TRPA [29, 66, 67], two walking only [52, 87], and four presented walking and cycling for commute separately [47, 57, 69, 72]).Of these, two studies reported that men were more likely to cycle for active transport compared to women [57, 69]. Dissimilarly, three studies found women had a higher probability of engaging in TRPA and a greater likelihood of high levels of active transport [46, 53, 74]; 21 studies observed no association to be present [14, 30, 32–35, 40, 45, 50, 54, 56, 58, 59, 63, 70, 71, 75, 78, 92–94].

Self-reported health status was assessed across eleven studies, five observed a significant, positive association with TRPA [35, 57, 74, 78, 94], one of which was longitudinal in nature [94]; a further six found no significant relationship [36, 41, 47, 50, 69, 75]. Eleven studies examined weight status; a statistically significant association was observed among five (six studies observed no significant association [35, 36, 45, 68, 75, 78]). Four studies found overweight and obese status was associated with increased odds of undertaking lower levels of TRPA (three cross-sectional [47, 69, 74], one longitudinal [95]) compared to healthy weight status, while one saw higher weight status was associated with greater TRPA in women living in socio-economically disadvantaged neighbourhoods [41]. Two studies found people who smoke had lower levels of TRPA [41, 45] compared to non-smokers while one cohort showed excessive alcohol consumption was associated with less engagement with TRPA [79]. Four studies observed no association between health behaviours (nutrition, smoking, and alcohol consumption) and TRPA engagement [47, 53, 57, 74].

While race or ethnicity was modelled as a covariate among many studies, fourteen articles examined its direct relationship with TRPA outcomes and only five [14, 47, 72, 79, 92] showed statistically significant associations, nine found no significant association [32, 35, 41, 46, 54, 59, 72, 75, 94]. Those who were non-white were more likely to undertake higher levels of TRPA [14, 47] than those who were white. Similarly, immigrant and minority populations were more likely to undertake TRPA [79, 92] than the remaining native residents. In a study from the US, white participants were more likely to undertake an active commute via bicycle compared to their Hispanic and African-American counterparts [72].

### Living arrangements

The living arrangements of participants (marital status, children and dependents in household, and pets) were assessed across 18 studies. Of the ten studies that considered marital status, four [29, 46, 53, 78] found that married and partnered individuals were less likely to engage in TRPA (for one study [53], in male participants only) than single people. A fifth study conversely found married individuals to have higher odds of undertaking TRPA [30] than singles, while seven studies showed no significant association [32, 35, 41, 45, 52, 57, 59]. An inverse association between the number of children/dependents in the household and the levels of TRPA was observed in two [30, 35] of seven studies. A third [47] found the presence of dependents within households of men of lower socio-economic status was associated with higher TRPA. Four studies found no association between dependents and TRPA [41, 45, 69, 92]. The presence of pets in the household was assessed in three studies [42, 44, 79], with only two finding significant association. One found that non-pet owners were more engaged in active commuting than pet owners [79]. A second study showed no significance of association [42]. The third study showed older adults that own and walk their dog had increased odds of walking for transport > 150 min/week, unlike dog owners who did not walk this dog whose odds of undertaking greater than 10 min of TRPA per week were greatly reduced [44].

### Socio-economic factors

Thirteen studies assessed employment status; six studies found employment status to be significantly associated with TRPA, seven observed no significant relationship [14, 29, 32, 36, 50, 68, 94]. Of these, four articles reported that being unemployed was associated with higher TRPA [45, 52, 61, 93] than being employed, while one – a study of women residing in lower-socio-economic neighbourhoods – found a positive relationship between employment and TRPA [41]. Increased odds of active commuting were present among those with the option of working from home and starting work during the hours of 11:00 to 15:59 compared with those that travelled to, and started work between the hours of 06:00 to 10:59 [72]. There were 19 studies that assessed the association between TRPA and individual and/or familial income (seven displayed non-significant relationships [37, 45, 53, 54, 58, 93, 94]). Eight studies observed a significant inverse association between income level and the amount TRPA performed [14, 34, 38, 46, 59, 72, 74, 92]. Two studies showed that increased household income was associated with an increased likelihood of engaging in TRPA compared with those with lower incomes [78, 87]. One study noted sex-based differences in associations with higher income in men yielding lower TRPA levels while higher income in women was positively associated with higher TRPA level [61].

Nine articles reported ten significant relationships between education level and TRPA, with conflicting results; a further 15 studies observed non-significant relationships [29, 30, 33, 45, 46, 52, 53, 58, 67, 69, 72, 74, 78, 87, 92]. Five studies (two longitudinal [62, 94]) found that higher levels of educational attainment were positively associated with higher levels of TRPA [38, 41, 62, 77, 94]. Conversely, three studies observed a negative association with individuals of the highest levels of TRPA having the lowest education levels [14, 34, 56], while one study found men of the lowest and women of the highest education levels were more likely to achieve high levels of TRPA engagement [61].

Greater socio-economic status (indicated by a range of proxy factors: education, employment, and income of the individual and those that also reside in their neighbourhood) was inversely associated with TRPA levels and odds of engagement in TRPA across nine studies [28, 47, 50, 56, 57, 63, 69, 75], only one study found no significant relationship [33].

Seven studies reported a significant negative association between motor vehicle access/ownership and the level of TRPA undertaken [14, 26, 29, 38, 45, 57, 78], an additional seven studies observed no-significant relationship [32, 35, 40, 66, 69, 92, 94]. Similarly, one study showed higher parking prices [59] to be associated with higher TRPA (one study reported non-significance [26]), while another found awareness of the negative consequences of car travel [66] to be associated with higher TRPA.

### Attitudes/beliefs/behaviours

Greater self-efficacy for active commuting was positively associated with TRPA across five studies [35, 41, 42, 62, 77], of which one was longitudinal [62]. Furthermore, six studies demonstrated that regular engagement, prioritisation, and enjoyment of physical activity was associated with higher TRPA [33, 36, 43, 66, 79, 95], three of these studies were of a longitudinal design [36, 43, 95]. A further eight studies found there to be no significant relationship between these PA behaviours and TRPA [26, 35, 42, 49, 53, 62, 68, 78]. Assessment of individual attitudes (e.g., perceived financial verses temporal costs [54]), found six studies to have no association [40, 41, 54, 66, 74, 77], whilst two observed a positive relationship. One study observed those who believed walking to be less convenient than motor vehicle transport were less likely to engage in TRPA [26], while individuals that perceived the number of immigrants residing in a neighbourhood to be high had higher odds of walking for transportation [82].

### Social exposures

When considering the association between social factors and TRPA, 11 significant and 17 non-significant associations between social support and modelling with TRPA were observed (see summary in Table 2). Feelings of trust and social cohesion among the neighbourhood was associated with higher TRPA in two studies (one cross-sectional [82], one longitudinal [62]), though was non-significant in nine studies [29, 40–44, 49, 57, 84]. Seeing others (pro-TRPA social modelling) such as family and friends undertake TRPA was positively associated with TRPA among three of seven studies [27, 49, 71], four observed no significance [35, 73, 77, 84]. Increased social support for TRPA (normalisation; from family, friends, co-workers or employers) was positively associated with higher TRPA among five studies [35, 41, 73, 77, 82]. Conversly, one cross-sectional study showed that family and friends suggesting more TRPA be undertaken was associated with reduced TRPA [49], while an additional three associations were non-significant [26, 66, 68]. It was suggested in one study that social norms related to cultural restrictions were associated with a lower level of TRPA among Pakistani women [52].

### Environmental exposures

A number of exposures related to commuter environment were associated with TRPA (Table 2). Eleven studies (including one of longitudinal design [95]) found that the odds of undertaking TRPA were higher among those who resided a shorted distance from their intended destination, with both perceived and objective distance of commute inversely associated with the level of TRPA undertaken [33, 50, 60, 63, 66, 69, 72, 77, 81, 91, 95]; one study observed no significant relationship [87].

Similarly, fourteen of sixteen studies found that a greater number of recreation, amenity, and retail destinations proximal to the areas of residence were associated with increased TRPA [27, 37–40, 44, 65, 71, 80, 81, 84, 85, 89, 91]; two studies observed no significant relationship [31, 93].

Fifteen studies examined the relationship between land-use mix (residential, commercial, and industrial co-location) and TRPA. Seven studies (two longitudinal [36, 62]) found positive associations between greater land-use mix and TRPA engagement [28, 36, 59, 62, 64, 70, 77]. Seven studies observed no significant association [51, 57, 58, 68, 89–91]. The final study found greater land use mix was associated with lower odds of active transportation [85]; however, as noted by authors, this study included industrial land use within its land-use mix metric – a value typically excluded due to its notable lack of association with PA outcomes and potential to influence associations.

Neighbourhood walkability was positively associated with TRPA in five studies [40, 59, 76, 78, 83] and was non-significantly associated among a further two [54, 65]. Of the twenty-one studies examining route connectivity, eleven (one longitudinal [95]) found areas with higher connectivity (intersections, cross-walks, destination accessibility) were associated with greater TRPA levels [24, 44, 49, 60, 70, 80, 84, 85, 89, 90, 95]. Ten studies observed no significant association [27, 31, 35, 37, 51, 58, 68, 77, 87, 93]. One study also observed connectivity to be positively related with TRPA amongt urban neighbourhoods, but not rural areas [80] while another conversly saw street connectivity to be associated with decreased odds of TRPA engagement [28].

Eight articles indicated that the presence of well maintained supportive infrastructure (such as curbing, bikelanes, bikepaths, and sidewalks bikepaths) was positively associated with TRPA [24, 25, 27, 37, 44, 71, 77, 89]. In contrast, one longitudinal study found older adults who perceived better infrastructure for walking had lower odds of engaging in TRPA compared to those perceiving worse infrastructure [62]. This contrasting finding may be because those spending greater periods undertaking TRPA within the neighbourhood may be more likely to observe a greater number of issues. A further nine studies observed there to be no signficant relationship present [28, 35, 47, 49, 57, 65, 68, 70, 95].

The relationship between public transport and TRPA was examined in 15 studies. A positive association was determined among 13 studies (one longitudinal [68]), reporting public transport proximal to residence and destinations resulting in higher TRPA [25, 38, 55, 57, 60, 65, 66, 68, 69, 80, 87, 89, 93]. However, one study found the number of bus stops and train frequency was negatively related to TRPA among low-income individuals only [59], a further study found no significant relationship [37]. Higher traffic levels were positively associated with TRPA levels in two studies [47, 69], though non-significant associations were observed among a further nine [24, 27, 30, 31, 35, 44, 50, 75, 95].

The density, greenspace, and landscape of the commuting environment was significantly associated with TRPA across 20 of the 42 relationships examined. Living in urban areas as opposed to rural areas was associated with increased TRPA in three studies (two cross-sectional [42, 72], one longitudinal [95]). Similarly, five studies found increased population and land density was associated with increased TRPA levels [14, 58, 64, 89, 92]. In contrast, two studies reported rural residents were more likely to undertake TRPA (compared with those from urban areas) [34, 41]; six found no significant relationship [53, 67–69, 74, 79]. One study found decreased housing and population density at the commute start point and higher density at the endpoint was associated with increased odds TRPA engagement [59], while 10 observed no significant association [28, 50, 51, 68, 70, 77, 87, 90, 91, 94]. Three studies observed that residing closer to green spaces and areas with greater tree-coverage was positively associated with TRPA [31, 60, 89]. Conversely, two studies found that individuals who resided in areas surrounded by buildings with less green spaces were more engaged in TRPA [79, 85], a further three observed no association [31, 44, 91]. One study found residents living neighbourhoods with flatter landscape were significantly more likely to walk for 150 min or more for transport per week [89], three found there to be no association [39, 44, 65]. Of the four studies examining park visitation, three observed non-significant relationships [37, 68, 91] whilst one demonstrated that increased park visitation was associated with greater odds of high TRPA levels [86]. Living closer to a river or coast was positively associated with TRPA in two studies [37, 89].

Perceived aesthetics of the environment was significantly associated with TRPA across 13 of 24 studies (14 relationships observed). Eleven of these studies reported that more attractive environments (free from litter and stray animals) were positively related to increased TRPA [24, 27, 39–41, 54, 60, 69, 70, 75, 77]. Two studies indicated different findings with one observing the aesthetics of an area was inversely associated with TRPA [28]. Another found that individuals with active occupations and high-levels of sedentary leisure time in areas of high pollution and low aesthetics had increased odds of high TRPA, while those with active leisure times travelling in low pollution and high aesthetics areas had increased odds of high TRPA levels [71]. Eleven studies found there to be no significant relationship between aesthetics and adult TRPA [25, 31, 37, 42, 44, 49, 50, 57, 62, 84, 95].

Weather was statistically signficantly associated with TRPA level in only one of three studies, though the magnitude of TRPA change was deemed to be clinically insignificant. Even after an extrapolation of effect, rain equating to ten inches during the travel day was associated with a decrease in walking for transport of just over half a minute on average per day, suggesting relative independance of weather and TRPA [48]. Two studies observed no significant relationship present between weather and TRPA [31, 35].

Neighbourhood and traffic safety were significantly associated with TRPA across 18 of 30 studies. Thirteen studies (one longitudinal [68]) showed greater perceived safety [24, 31, 42, 44, 47, 57, 68, 70, 75, 77, 84, 93], lower crime rates [31], and perceived safety from traffic (including visibility, safe traffic speeds, and safe road crossings) [24, 30, 44, 70, 77, 93] were positively associated with TRPA. Five studies observed greater perceived safety from crime, stray animals, and traffic were associated with lower TRPA [28, 39, 54, 62, 80], one of which was longitudinal [62]. Twelve studies observed no association between safety and adult TRPA [25, 27, 29, 35, 37, 41, 43, 49, 78, 88, 91, 95]. A higher presence of streetlighting was positively associated with greater levels of TRPA among seven [39, 44, 80, 81, 88, 89, 91] of ten studies (three non-significant [25, 31, 60]).

## Discussion

This is the first comprehensive synthesis of the correlates and determinants of TRPA among adults. In this systematic review, findings from multiple disciplines of research across the past decade were used to identify a small number of factors that demonstrated consistent associations with adult TRPA and a large number of factors that exhibited inconsistent relationships. Thirty-six factors were assessed across the 73 studies included in this synthesis, with seven factors consistently associated with adult TRPA: socio-economic status, self-efficacy, social normalisation, distance of travel, destination, public transportation, and the presence of streetlighting. These factors represent all layers of the social-ecological model (individual, social, and environmental), highlighting the multi-layered nature of the influences of adult TRPA. This study acts to highlight these 36 factors as variables for consideration in the development of future framework while also bringing attention to the need for further longitudinal and multidisciplinary studies.

### Individual level factors

Nineteen individual level factors assessed as potential correlates and determinants of adult TRPA were identified, including age, sex, health, health behaviours, living arrangements, socio-economic circumstances, and attitudes and beliefs. However, only two (individual socio-economic status and self-efficacy) were consistently associated with adult TRPA outcomes.

Socio-economic status was assessed across studies via differing combinations of education, employment, and income (both of the individual and those that also reside in their neighbourhood). Eight of nine studies found higher socio-economic status to be associated with lower levels of TRPA. Association between socio-economic status and PA has also been observed in the domain of leisure-time PA. This mutual correlate could be due to the shared discretionary nature of these types of PA [96]. However, literature has shown self-efficacy to mediate the relationship between PA and individual- and area-level income and education [97]. Moreover, it must be acknowledged that for some, active commuting may be a necessity rather than a choice. Higher TRPA observed among those of lower socioeconomic position may be due to costs associated with purchasing and running a car (e.g., servicing, registration, parking) leading to higher reliance on other forms of transportation, such as public transport, walking, and cycling [98]. These findings suggest that those of higher socio-economic status provide a low TRPA population to which interventions may be targeted.

Self-efficacy for active commuting was also identified as a consistent correlate of adult TRPA. Self-efficacy refers to an individual’s judgement of their capability to organise and integrate TRPA behaviours into their lifestyle. As a discretionary domain of physical activity, the association between greater self-efficacy for active commuting and higher adult TRPA engagement unsurprisingly mirrors that of leisure-time PA [96]. Furthermore, self-efficacy has been observed to affect the amount of effort devoted to a task, and the magnitude and length of persistence when difficulties are encountered [99], therefore, affecting engagement as well as TRPA levels and maintenance. These findings are important as they highlight the need for policymakers to not only provide infrastructure to facilitate TRPA, but also to develop strategies that work to engage and encourage individuals so that the TRPA infrastructure provided will be used.

### Social level factors

Few social-level factors were examined (*n* = 3) and even fewer were associated with TRPA. No association was observed between social cohesion and TRPA, and associations between social modelling and TRPA were equivocal. Only social normalisation was observed as a consistent correlate of greater TRPA among adults. Often the normalisation of TRPA was experienced via the implementation of pro-TRPA policies in the workplace and peers and family voicing their support of TRPA practices. Some contrasting associations were found between normalisation and TRPA engagement. It is possible that findings of decreasing TRPA despite greater encouragement from family and friends [49] may be present only due to reverse causality (e.g., those with lower TRPA receiving greater encouragement) and cross-sectional assessment [49]. Prior studies have suggested that interventions aimed at normalising the act (TRPA) as well as its associated factors may lead to greater TRPA [100]. Hence, further study into social attitudes towards these associated factors may provide a greater understanding of the social structures governing TRPA performance and highlight points for future intervention.

Few studies reported significant associations between social factors and TRPA outcomes compared with literature examining leisure-time PA. This may be attributable to the necessity of travel in today’s society. While leisure-time PA and TRPA share a discretionary nature, feelings of social cohesion and positive modelling may encourage society members to undertake leisure-time PA. However, those without the capacity to undertake private transportation or those with greater self-efficacy for TRPA may undertake an active commute irrespective of their social or physical environment – an important consideration when tailoring domain-specific interventions.

A distinct lack of longitudinal analyses of TRPA and social factors (n = 3) was also highlighted. Failure to examine longitudinal relationships between social-level factors and TRPA prevents the ascertainment of temporality (i.e., determination of whether the levels of TRPA observed were obtained before introduction to the social environment or whether TRPA levels were the result of the relationship between the social environment and the individual). Resultantly, a gap remains surrounding the relationships of social factors (i.e., policy, positive TRPA modelling and normalization, and social cohesion) with adult TRPA outcomes. As highlighted by leisure-time PA [101], these factors have the potential to act as independent determinants of TRPA engagement, and therefore warrant further investigation. Due to the unique circumstances afforded via the international coronavirus disease (COVID-19) pandemic, there is potential to interpret the results of natural experimentation in which the relationship between social cohesion and the uptake of public transportation and TRPA is observed following the reduction and cessation of COVID-19 restrictions.

### Environmental level factors

Eighteen environmental-level factors were assessed including sidewalks, supportive infrastructure, land-use mix, traffic, and weather. However, only four environmental correlates and determinants of adult TRPA were identified: distance travelled, concentration/number of destinations, public transportation access, and the presence of streetlighting.

As previously established, greater distance of travel was consistently associated with lower TRPA levels and engagement [102, 103]. TRPA engagement was higher among those who resided closer to their intended destination, with increased distance of commute inversely associated with the level of TRPA undertaken. Additionally, destination concentration was positively associated with adult TRPA. Those residing and travelling among areas with a higher number of destinations (i.e., amenity, retail, and recreation centres) in close proximity to commute route and residence observed higer levels of TRPA. Public transport was also identified as a correlate and determinant of adult TRPA. A positive relationship was observed, with greater public transport frequency and higher number of public transport terminals more proximal to the route start and destination associated with higher levels of TRPA. These findings may be based upon principles of convenience, with observations surrounding public transport accessibility and TRPA outcomes similar to those observed with distance and destination. These findings suggest that urban and transport planning (centred upon the creation of destinations within both a walkable distance of the home and a comprehensive public transport network) has the potential to encourage TRPA engagement and facilitate the achievement of recommended PA levels.

A greater presence of streetlighting was associated with higher TRPA levels. The presence of streetlighting has the potential to facilitate greater levels of active commuting by allowing individuals to better navigate their route during periods of darkness. Furthermore, literature suggests that the presence of streetlighting yields higher levels of perceived safety [104]. Though not shown to be consistently associated with TRPA in this review, increased safety of the commute route has the potential to relate with commute habits when adjusted for additional factors such as age, sex, socio-economic status, and self-efficacy. As such, the installation of streetlighting along commuter routes may be seen as a key means of increasing TRPA engagement among those required to commute during periods of darkness.

Studies of the built environment (land-use mix, population and residential density, walkability, connectivity, supportive infrastructure, and urban/rural status) and adult TRPA were equivocal and inconclusive. Similarly, relationships between TRPA and the natural environment (i.e., greenspace, proximity to water bodies such as rivers and coast, and gradient) yielded equivocal and inconsistent results. This suggests that unlike leisure-time PA [105], TRPA may be more dependent on where, how, and how far an individual is travelling, rather than the landscape in which the commute occurs. This further highlights the need for TRPA intervention design to be considered separately to those of the leisure-time PA domain.

### Limitations and strengths

Only English language, peer-reviewed studies from the last decade were included in this systematic review. Thus, grey-literature, non-English studies, and literature published prior to our cut-off were not included. As many exposures and outcomes across studies were heterogeneous in their measurement techniques, meta-analysis was not appropriate and therefore, quantitative estimates of associations could not be presented; we recommend future studies consider meta-analysis if appropriate All screening was performed by two authors independently, thus minimising selection bias and improving reliability of the screening process [106]. Among the studies included in this review, most focussed on assessing TPRA using single-discipline lenses; few studies employed multi-disciplinary frameworks. Comprehensively assessing multi-level and/or multi-disciplinary models has the potential to lead to identification of novel combinations of individual, social, and environment exposures that cannot be identified in single-discipline or single-population studies [107]. In turn, this could facilitate the formation of tailored interventions with increased effectiveness.

Self-report of both exposures and outcomes amongst studies is of potential methodological concern due to the possibility of recall or social desirability biases. This potential for recall bias was lessened via assessments of quality that ensured studies with high risk of bias and lower quality were excluded from this review. Furthermore, TRPA assessment via questionnaire has been found to be a valid and reliable form of measurement [108]. While objective assessment of TRPA by accelerometer is possible, it still relies on self-report of movement during the day to attribute the collected data to a specific PA domain [109]. Studies were undertaken in different countries; thus, findings of included studies may differ due to being shaped by different cultural beliefs around TRPA promotion, differing infrastructure standards and varied social and individual beliefs. This may be illustrated within this review via the identification of societal norms as potential factors responsible for sex-based disparities in the TRPA of Pakistani participants [52]. However, the multi-national nature of this systematic review is also a strength, providing insight and further generalisability into the relationships identified. Additionally, the varying sample sizes of studies included may have resulted in studies with large samples observing significant relationships for some factors, while studies with lower participant numbers and statistical power may have found non-significance. This may have resulted in this review misclassifying associations as inconsistent. However, only 25 studies had a sample size less than 1000 of which 4 had a sample size less than 300, suggesting statistical power is unlikely to explain the observed findings. Most studies (94.5%) included in this review measured TRPA for any purpose, but four only considered TRPA for work/school purposes. While this is a potential limitation, particularly for those who are not employed or in education, the small number of these studies are unlikely to impact on the overall findings. Further, in studies examining sex and age for example, the minimum, maximum and median sample sizes did not markedly differ according to direction (positive, negative, null) of association (see Additional file 3). This study guides future analyses by presenting all observed factors and highlighting inconsistencies of association, so that future researchers do not fail to consider key covariates when literature searches to inform model formation suggest non-significant association.

Furthermore, the multi-disciplinary nature of this review, and its use of a social-ecological model provides a diverse series of factors organised within a well-established theoretical framework. However, it must be noted that factors from within the organisational and policy levels of the social-ecological model were not identified within studies included in this review and warrant investigation in future research. Finally, the 73 published studies compiled within this review provide a considerable catalogue of literature that acts to strengthen our findings.

### Future directions

This review identifies a number of future research directions. There remains a substantive gap in the literature on longitudinal relationships with adult TRPA outcomes – as highlighted by the very low number of longitudinal studies identified in this review (*n* = 7). While cross-sectional studies allow for the assessment of correlation, a temporal relationship cannot be inferred, thus preventing insights into causality. This absence of longitudinal studies may be due to the high monetary, temporal, and resource expenses associated with this mode of observation. To determine whether TRPA is an action brought about by the current needs and circumstances of the individual or a learnt behaviour, further longitudinal research is needed. The longitudinal assessments included in this review examined a range of factors associated with TRPA across a number of different stages of adulthood. However, failure to incorporate factors from a range of social-ecological levels may have limited their findings. For example, the use of perceived environmental measures instead of objective assessments has the potential to reduce the magnitude of association between built environment and TRPA. This is because perceptions represent the subjective interactions between an individual and their environment (e.g., an individual of lower self-efficacy or poorer health may not believe their environment is conducive to TRPA, while another more motivated or physically able individual may find the same environment to be favourable for active commuting) rather than objective assessments of the built and natural environment (e.g., distance of route, or the presence of streetlighting and supportive infrastructure). Similarly, additional longitudinal studies within this review examined the built environment with adjustment for individual-level socio-economic factors only. By overlooking the potential role of social factors (such as social support) and individual level cognitions (such as beliefs or motivation), these studies may under- or over-estimate associations. As such, it is recommended that future longitudinal analyses would benefit from combined analysis or adjustment for both objective and perceived measures, as well as a focus on better encompassing a range of factors spanning the social-ecological model. Future research could assess tracking and patterns of both TRPA and its associated factors across the life-course. Further, randomised controlled trials testing interventions to increase TRPA are warranted, particularly assessing means of increasing efficacy, and participation in active commuting on routes where distances may be greater and destinations more sparce (previously observed to be associated with decreased TRPA). This may be via changes in policy and practice that ultimately normalise and promote public transport and TRPA. These studies could prove impactful among those of higher socio-economic status who have been identified as undertaking lower levels of TRPA.

At present, there has been greater examination of the environmental and individual-level correlates and determinants of TRPA compared with those of social factors. Further study of the social factors that associate with TRPA is required to bring TRPA research into line with literature of other PA domains. Furthermore, this review observed an absence of factors from organisational and policy levels of the social-ecological model. This finding highlights a need for further analysis of how organisational and policy-based factors relate to TRPA outcomes.

Future studies should carefully model the associations between exposures and TRPA considering the potential for confounding, mediation, and effect modification between exposures across the socio-ecological model. This may identify potentially modifiable factors to target to increase TRPA among certain groups, for example women or those in rural areas. Examination of multi-level pathways and mediatory relationships are required to provide insight into the underlying mechanisms through which TRPA may be promoted and subsequently increased.

## Conclusion

This systematic review provides a synthesis of correlates and determinants of TRPA from English peer-reviewed literature of the last decade. Spanning multiple disciplines of research, findings were presented within a social-ecological framework, forming a comprehensive resource to inform future studies and interventions. While socio-economic status, self-efficacy, social normalisation, distance of travel, destinations, public transportation, and the presence of streetlighting were consistently associated with adult TRPA, all factors observed to be associated with TRPA in this review could be considered for inclusion within prospective analyses. Future studies that consider potential mechanisms previously overlooked due to the single-disciplinary nature of prior research may provide a greater understanding of factors amenable to intervention. Those developing policies and strategies to increase TRPA should consider factors at the individual, social, and environmental level, as well as the potential interactions amongst these factors, to maximise the likelihood of effectiveness.

## Supplementary Information

**Additional file 1.** Modified Newcastle-Ottawa quality assessment scale. Modified Newcastle-Ottawa Scale used in the assessment of article quality.

**Additional file 2.** Article quality assessment table. Quality assessment table for articles included within systematic review.

**Additional file 3.** Sample size distributions. Sample size distribution for TRPA’s relationship with age and sex.

## Data Availability

All data generated or analysed during this study are included in this published article (and its supplementary information files).

## Abbreviations

EPAQ2: European prospective investigation into cancer and nutrition Physical Activity Questionnaire version two
GPAQ: Global Physical Activity Questionnaire
IPAQ (-L/-S): International Physical Activity Questionnaire (-Long / -Short version)
NPAQ: Neighbourhood Physical Activity Questionnaire
MeSH: Medical subject heading
MET: Metabolic Equivalent of Task
MOOSE: Meta-analyses and systematic reviews of observational studies
PA: Physical activity
PRISMA: Preferred reporting items for systematic reviews and meta-analyses
PROSPERO: International prospective register of systematic reviews
STAQ: Sedentary and Transport Activity Questionnaire
SQUASH: Short Questionnaire to Assess Health-enhancing Physical Activity
TRPA: Transport-related physical activity
