# Supplementary material for: Correlates and determinants of transport-related physical activity among adults: an interdisciplinary systematic review
Source: BMC Public Health. 2022 Aug 10;22:1519. doi: 10.1186/s12889-022-13937-9 (PMC9363261; doi:10.1186/s12889-022-13937-9)
Supplement: Supplementary file 1 — Additional file 1. Modified Newcastle-Ottawa quality assessment scale. Modified Newcastle-Ottawa Scale used in the assessment of article quality. [file 12889_2022_13937_MOESM1_ESM.docx]

**Modified Newcastle - Ottawa Scale for the assessment of study quality.**

Criteria as shown when undertaking quality assessment in Covidence.

**SELECTION 1 - Representativeness of the sample**

**Truly*** = "Truly representative of the average in the target population (all subjects or random sampling)*

**Somewhat*** = Somewhat representative (non-random sampling)*

**Unrepresentative** = Selected group / no description of the derivation of the cohort

**SELECTION 2 - Sample size**

**Justified*** = Justified and satisfactory *

**Not Justified**

**SELECTION 3 - Non-respondents**

**Satisfactory*** = Comparability between respondents and non-respondents’ characteristics is established, and the response rate is satisfactory*

**Unsatisfactory** = The response rate is unsatisfactory, or the comparability between respondents and non-respondents is unsatisfactory

**No description** = No description of the response rate or the characteristics of the responders and non-responders

**SELECTION 4 - Ascertainment of the exposure**

**Validated**** = Validated measurement tool used **

**Non-validated*** = Non-validated measurement tool used, but is available or described*

**No description** = No description of the measurement tool

**COMPARABILITY - The subjects in different outcome groups are comparable, based on the study design or analysis. Confounding factors are controlled.**

**Comparable*** = Subjects are comparable and the study controls for or assesses compulsory covariates including age and sex.

**Not comparable**

**OUTCOME 1 - Assessment of the outcome**

**Independent blind assessment ****

**Record linkage****

**Self-report***

**No description**

**OUTCOME 2 - Statistical test**

**Appropriate*** = The statistical test used to analyse the data is clearly described and appropriate, and the measurement of the association is presented, including confidence intervals and/ or the p value*

**Not appropriate** = The statistical test is not appropriate, not described, or incomplete

**OUTCOME 3 - Longitudinal assessment only**

**Complete participant follow-up*** = Complete follow-up/ all subjects accounted for*

**Acceptable participant follow-up* =** Subjects lost to follow-up unlikely to introduce bias. Number lost is less than or equal to 20%, or the description of those lost suggests no difference from those followed*

**Insufficient follow-up** = Follow-up rate less than 80% and no description of those lost

**No statement**

**Not applicable**

**FINAL QUALITY RATING**

Maximum score for cross-sectional study = 9*

Maximum score for longitudinal study = 10*

Criteria for quality rating:

**Good** (high) ≥ 7*

**Fair** (moderate) = 5* and 6*

**Poor** (low) ≤ 4*

N.B. Poor quality studies will be excluded
