# Supplementary material for: Correlates and determinants of transport-related physical activity among adults: an interdisciplinary systematic review
Source: BMC Public Health. 2022 Aug 10;22:1519. doi: 10.1186/s12889-022-13937-9 (PMC9363261; doi:10.1186/s12889-022-13937-9)
Supplement: Supplementary file 3 — Additional file 3. Sample size distributions. Sample size distribution for TRPA’s relationship with age and sex. [file 12889_2022_13937_MOESM3_ESM.docx]

**Additional file 3 - Sample size distribution for TRPA’s relationship with age and sex.**

The number participants in studies found within each of Table 2’s positive, negative, and non-significant categories were reviewed. The distribution of study sample sizes were found to be acceptable (large sample sizes are not observed in one category while small are in another). Examples of sample size distributions for the 39 relationships between age and TRPA, and 33 relationships between sex and TRPA are shown within Table A3.1 and A3.2

| Table A3.1 - Distribution of sample sizes among studies with positive, negative, and non-significant relationships between age and transport-related physical activity | | | |
| --- | --- | --- | --- |
|  | Positive  (3 studies) | Negative  (12 studies) | Non-significant  (24 studies) |
| Minimum, n | 4349 | 441 | 161 |
| Median, n | 15105 | 1304 | 1137 |
| Maximum, n | 173206 | 11035 | 308901 |
|  | | | |

| Table A3.2 - Distribution of sample sizes among studies with positive, negative, and non-significant relationships between sex (male) and transport-related physical activity | | | |
| --- | --- | --- | --- |
|  | Positive  (9 studies) | Negative  (3 studies) | Non-significant  (21 studies) |
| Minimum, n | 441 | 1720 | 311 |
| Median, n | 1833 | 5180 | 1111 |
| Maximum, n | 152573 | 7280 | 308901 |
|  | | | |
